# Predictive Value of Positive Endocervical Curettage Results Obtained During Colposcopy

**DOI:** 10.3390/diagnostics16070976

**Published:** 2026-03-25

**Authors:** Julia Wittenborn, Cangül Cuma, Lieven N. Kennes, Laila Najjari, Elmar Stickeler, Tomas Kupec

**Affiliations:** 1 Department of Obstetrics and Gynecology, Center for Integrated Oncology (CIO Aachen, Bonn, Cologne, Düsseldorf), University Hospital of Rheinisch-Westfälische Technische Hochschule Aachen (RWTH), 52074 Aachen, Germany; 2 Department of Economics and Business Administration, University of Applied Sciences Stralsund, Zur Schwedenschanze 15, 18435 Stralsund, Germany

**Keywords:** endocervical curettage, HSIL, LEEP, HPV, cervical cancer screening

## Abstract

**Objective:** The aim of this study was to evaluate the positive predictive value of endocervical curettage (ECC) during colposcopic examination in a dysplasia unit since the implementation of the new cervical cancer screening program in Germany (January 2020). **Methods:** A total of 202 patients who presented for colposcopy at the Dysplasia Unit of the University Hospital Aachen, Germany, between January 2020 and October 2023, who had cervical intraepithelial neoplasia 1+ (CIN1+) in the endocervical curettage and received a loop-excisional procedure of the cervix (LEEP), were included in a retrospective cohort analysis using machine learning techniques (random forest analysis and leave-one-out cross-validation). **Results:** There was a low agreement between the results of the ECC and the CIN status after LEEP (kappa 0.0239). A positive result in the histological specimen of the excisional procedure of the cervix (CIN2+) was obtained in 73.7% in case of CIN1 detection in the ECC, 69.4% in case of CIN2 detection in the ECC, and 80.6% in case of CIN3 in the ECC. In the multivariate analysis, the result of colposcopy and the transformation zone, especially combined (kappa 0.259, *p* = 0.0004), had the highest predictive value with regard to the CIN status. **Conclusions:** ECC is associated with a low agreement rate in comparison to the final histological result of the LEEP, which should be kept in mind when counseling patients. A finding of CCIN1in an ECC obtained during colposcopy following abnormal HPV-based cervical cancer screening results should be considered an indication of a possible intracervical dysplasia.

## 1. Introduction

Cervical cancer is one of the most common cancers in women. Worldwide, approximately 660,000 women were diagnosed with cervical cancer in 2022 and 350,000 women died [1]. In Germany in 2020, 4640 women were diagnosed with cervical cancer and 1546 women died from it [2]. Since the introduction of the cervical cancer screening program in the 1970s, using cytological smears, this tumor has become a less frequent entity in Germany [2].

Due to overwhelming evidence from long-term prospective cohorts and randomized clinical trials demonstrating that high-risk human papillomavirus DNA (hrHPV DNA) testing is considerably more sensitive than cervical cytology for the detection of cervical intraepithelial neoplasia grade and cancer, a new cervical cancer screening algorithm was implemented in 2020 [3,4,5,6,7]. Precancerous lesions such as high-grade squamous lesions (HSIL) are typically caused by persistent infection with human papillomavirus (HPV) and can progress to invasive cervical cancer. Detecting HSIL therefore plays a crucial role in cervical cancer prevention [8].

In Germany, hrHPV testing was introduced as a supplementary test in patients aged 35 years and older in 2020 [9]. As hrHPV testing is widely used, the optimal clinical management of women with hrHPV infection, especially combined with no or minor cytological abnormalities, remains a challenge. According to the German national guidelines, patients with suspicious cytological results are referred for colposcopic examination [9]. Additionally, patients with one-time low-grade cytologic abnormalities (ASC-US) are referred to colposcopy if they carry a hrHPV infection. Also, patients with persistent hrHPV infection over one year and normal cytology (NILM) are referred. As HPV infections occur at the transformation zone (TZ) of the cervix, which is the squamocolumnar junction between the endo- and ectocervix, the colposcopic examination focuses on this area. Depending on the visibility of the squamocolumnar junction, the transformation zone (TZ) is categorized into three types: type 1 is completely visible, type 2 is completely visible after splaying of the cervix, and type 3 (T3) is not completely visible [10,11].

TZ T3 is a constitutional finding, predominantly determined by hormones, obstetric history, and the lack of estrogens, representing a common situation during menopause. TZ T3 is related to controversies in colposcopy patient management, requiring skilled physician input and often a multidisciplinary approach. With the aging population globally, it is anticipated to represent a growing issue in the forthcoming decades. As in the case of a type 3 transformation zone (TZ), only limited assessment of the transformation zone is possible; this may result in an increased risk of missing disease by up to twofold [12,13]. To improve the detection and reduce the risk of missing disease in this group of women, endocervical curettage (ECC) can be performed but remains controversial [14,15].

Here, we present a study evaluating the predictive value of a positive ECC during colposcopic workup of patients in the cytology and HPV-based screening program. In contrast to most other published studies concerning ECC, we focused on the diagnostic accuracy of the performed ECC. All HSIL diagnoses that were obtained in the cervical dysplasia unit using ECC were matched with the results of the afterwards performed excisional procedure.

Additionally, we sought to determine the best predictive parameters for the presence of cervical intraepithelial neoplasia 2+ (CIN2+) in this cohort using multivariate random forest modeling.

## 2. Materials and Methods

### 2.1. Study Population

The retrospective cohort analysis included 202 patients between the ages of 23 and 81 years who presented for colposcopy due to abnormal cytological smears or persistent hrHPV infection at the Dysplasia Unit of the University Hospital Aachen from January 2020 to October 2023 and underwent ECC.

### 2.2. Data Collection

The patients that presented to the Dysplasia Unit of the University Hospital Aachen underwent a standardized examination at the certified dysplasia unit. After the patients were positioned adequately, a colposcopic examination was performed using a Leisegang 3MCV colposcope (Fa. Leisegang Feinmechanik Optik GmbH, Berlin, Germany). Every patient that underwent an examination, first received a control cytology by taking a Pap smear of the cervix and a test for human papillomavirus (PCR for HPV DNA). The cytology upon referral as well as the control cytology were divided into PAPI/II (which correlates with NILM in the Bethesda classification), PAPIIID1 (LSIL), PAPIID2/p/g (HSIL; ASC-H, AGC endocervical, favor neoplastic), PAPIVa-p (HSIL), and PAPIVb-p/V (HSIL). The classification of the HPV to the different categories was in accordance with the International Association of Cancer Registries (IACR) guidelines. Category 1a included HPV 16 and 18. HPV 31/33/35/52/58/45 were comprised in category 1b. Category 2 included HPV 51/56/39/59 and category 3 HPV 68/73/66.

Subsequently, a 5% acetic acid solution was applied to further examine the transformation zone. The transformation zone was categorized in one of three different categories depending on the visibility of the squamocolumnar junction: type 1 was completely visible, type 2 was completely visible after splaying of the cervix, and type 3 (T3) was not completely visible. Correspondingly, colposcopic findings were assigned regarding the nomenclature of the IFCPC (International Federation for Cervical Pathology and Colposcopy) from 2011. As shown in Table 1, the colposcopic findings were divided into normal, such as ectopy, atrophic epithelium, or cysts and minor changes, including for example, fine mosaicism or fine punctation. Major changes, such as coarse mosaicism or punctation, inner border sign, ridge sign and even colposcopic findings hinting at possible invasion—for instance atypical vessels, exophytic growth of lesions and necrosis—were taken into consideration [11]. The main inclusion criterion for the study was a positive result of the performed ECC. As any suspicion in the ECC should lead to a diagnostic loop-excisional procedure according to the German guidelines, all patients with a cervical intraepithelial neoplasia as a result of the ECC were included (CIN1, CIN2 and CIN3). For the analysis, patients were divided into two subgroups: Patients with a positive histological result only in the ECC with no colposcopy-directed biopsies or colposcopy-directed biopsies with normal histologic result comprised the *ECC-only* group. Patients with a positive histological result in both ECC and colposcopy-directed biopsies were comprised in the *ECC + biopsy* group. All included patients received a loop-excisional procedure of the cervix. All operations were performed under colposcopic view of the cervix and were carried out by experienced, highly qualified and certified staff at the certified colposcopy unit of University Hospital Aachen under general anesthesia. Decisions regarding surgical treatment were based on the German guideline for the prevention of cervical cancer. The primary endpoint of the analysis was the cervical intraepithelial neoplasia (CIN) status after loop-excisional procedure (LEEP) of the cervix, which was further divided into positive (CIN2+) and negative (CIN1, dysplasia-free).

### 2.3. Ethical Approval

Prior to the beginning of the retrospective study, it was approved by the Ethics Committee of the RWTH Aachen University Faculty of Medicine in June 2024 (EK 24-196). Subsequently, this retrospective study was executed on the basis of the ethical standards of the 1964 Helsinki Declaration and its later amendments.

### 2.4. Statistical Analysis

The statistical analysis included descriptive, bivariate, and multivariate analysis methods. Continuous variables were described as mean ± standard deviation (SD) and minimum to maximum (Min–Max). Categorical variables were presented as absolute frequencies and percentages.

For the bivariate analysis, continuous variables were examined by comparing means and standard deviations, and categorical variables were examined using contingency tables. To analyze the agreement between the result of the colposcopy-directed ECC and the result of LEEP, Cohen’s Kappa coefficient and plain agreement were calculated. A Cohen’s Kappa close to 1 indicates strong agreement beyond chance; a value close to 0 indicates random agreement only. For the multivariate analysis, random forest models were used to describe and evaluate predictor variables for the binary outcome. Random forest methods are machine learning algorithms that are able to flexibly model complex nonlinear relationships and high-order interactions among predictors while remaining robust to multicollinearity and overfitting. Weighted random forest models were employed to account for class imbalance in the binary outcome and to improve predictive performance and variable importance estimation for the underrepresented outcome class (non-CIN2+). Model performance was assessed using leave-one-out cross-validation (LOOCV). In this approach, each observation was iteratively held out once as a test case, while the model was trained on all remaining observations. The trained model was then used to generate a prediction for the held-out observation. This process was repeated until every observation had served as the test set exactly once, ensuring that all performance estimates were derived from out-of-sample predictions. LOOCV was chosen to maximize the use of available data for model training while providing an unbiased estimate of predictive performance, which is particularly advantageous in studies with limited sample sizes.

## 3. Results

**Figure 1:**
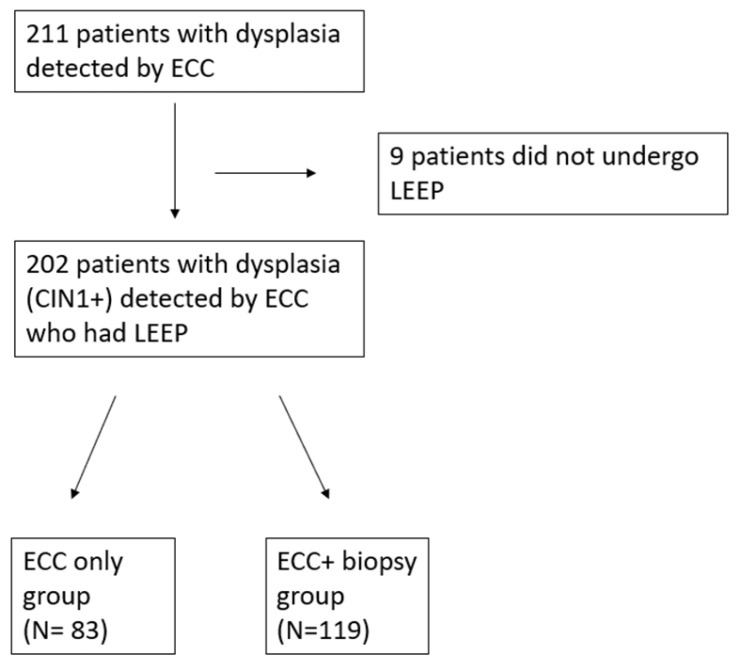
Patient flow chart.

In total, 211 patients with dysplasia detected by ECC were assessed for eligibility. Patients who did not undergo a loop excision, or those for whom the results of the loop excision were not available, were excluded. A total of 202 patients were included in the final analysis (see Figure 1).

The mean age of all included patients was 43.61 years (SD: 11.01), with a minimum age of 23 and a maximum age of 81. Smoking status was recorded for 77.7% of all the included patients; 60.4% were smokers, whereas 17.3% were non-smokers, and in 22.3% the smoking history was unknown. The distribution of cytological results upon referral is displayed in Table 1, as well as the control cytology, the result of the colposcopy, and the localization of the transformation zone. A total of 50% of the patients included had a T3 transformation zone. A total of 96,5% of the patients had HPV high-risk infection; in 36.6% of the patients, multiple HPV high risk viruses could be detected. The performed ECC showed CIN1 in 9.4% (*N* = 19), CIN2 in 24.3% (*N* = 49), and CIN3 in 66.34% (*N* = 134). In 81.7% of the included patients, cervical biopsies were taken in addition to the performed ECC. The results of the colposcopy-directed biopsies are displayed in Table 1. All included patients received a loop-excisional procedure of the cervix. In 58%, CCIN3was detected in the excisional specimen, in 18% a CCIN2 and 12% had CIN1. In 11%, the specimen was free of dysplasia.

Table 2 shows the results of the bivariate statistical analysis using CIN2+ (result of LEEP) as the positive endpoint. In patients with a negative endpoint (non-CIN2+), the mean age was 46 years, whereas the patients’ mean age was 43 years in the CCIN2 group. Of 33 patients with normal colposcopy, 20 (60.6%) had CIN2+ as the result of the excisional procedure of the cervix. Of the 32 included patients with normal cytology upon referral (PAP I/II), CIN2+ was detected in 22 (68%).

The agreement between the result of the ECC and the histological specimen of the LEEP is displayed in Table 3. A positive result in the histological specimen of the excisional procedure of the cervix was obtained in 73.7% of cases with CIN1 detection in the ECC, 69.4% with CIN2 detection in the ECC, and 80.6% with CIN3 in the ECC. A total of 22.4% of the patients with CIN2+ in the ECC showed a CIN1 or a dysplasia-free result after LEEP. The plain agreement between the histological result of the ECC and the result of the LEEP for the binary endpoint (CIN2+ vs. non-CIN2+) was 0.73. Cohen’s Kappa for this agreement is 0.0239.

In Table 4 the agreement between the histology of the ECC and the result of the LEEP is displayed for the different subgroups. In *N* = 83 cases, no additional colposcopy-directed biopsies were taken (“ECC-only group”). A positive histological result in the excisional procedure was found in five out of ten cases (50%) with CIN1 in the ECC (“ECC-only group”). In the ECC + biopsy group, a positive histology was found in all nine patients (100%) with CIN1 in the ECC.

For the multivariate analysis, several machine learning models (weighted random forest—LOOCV) were evaluated using different parameter configurations. The best-performing model, which accounted for class imbalances, included the colposcopy and the transformation zone. This model achieved an accuracy of 0.6671 and a balanced accuracy of 0.6671, both on unseen data as assessed by LOOCV. Even when correcting for random agreement, a relatively fair agreement remained with κ = 0.2588 (*p* = 0.0004).

## 4. Discussion

This retrospective single-center study included 211 colposcopies with histology samples obtained using ECC. In 202 cases, the result of the excisional procedure was available. ECC is a low-risk, low-morbidity procedure that is widely available and also time- and cost-effective. According to the German guidelines, it is recommended to perform an ECC in cases of a T3 transformation zone. According to the 2017 Colposcopy Standards Consensus Guidelines of the American Society for Colposcopy and Cervical Pathology (ASCCP) and a recent update article, ECC is recommended in several patient scenarios, such as when CIN2 is detected, to rule out intracervical CIN3+, and to determine whether a conservative or a surgical approach may be feasible; when there is a known hrHPV type 16 or 18 infection; abnormal cytology in patients with a history of HSIL or carcinoma; suspicion of CCIN3 adenocarcinoma in situ (AIS) or invasive cancer; the presence of TZ3 at colposcopy, and others [16]. The guidelines state that, although ECC is obsolete in pregnant patients, it is acceptable for all nonpregnant patients undergoing colposcopy [16]. Data on routinely performed ECC is inconclusive. Studies suggest that the rate of additional diagnosis of CCIN2 by means of ECC is between 1 and 13% [17,18]. The benefits of ECC may be greater in older women [16,17]. Other studies report that changes in treatment plans on the basis of additional ECC are only made in a small group of patients [17,19].

Including a large number of patients with a positive ECC and a subsequently performed loop-excisional procedure, the accuracy and predictive value of the performed ECC could be evaluated in the presented study. We observed a positive histological result after LEEP (CIN2+) in 73.7%, 69%, and 80% of the cases with CIN1, CIN2, and CIN3 in the ECC, respectively. A total of 22.4% of the patients with CIN2+ in the ECC showed only CIN1 or no dysplasia in the LEEP specimen (Table 3). Even though the plain agreement was 72.7% (non-CIN2+ vs. CIN2+), correcting for random agreement, Cohen’s Kappa was low.

The agreement was lower in the “*ECC-only*” subgroup (Table 4) than in the “ECC + biopsy” group. Here, 35.6% of the patients with a CIN2+ in the ECC during colposcopy had normal histological results in the excisional procedure. Conversely, 20 of 33 (60.6%) patients with normal colposcopy and CIN1+ in the ECC had a CIN2+ as the result of the excisional specimen of the cervix. Among the 32 included patients who had normal cytology upon referral (PAP I/II), CIN2+ was detected by ECC in 22 (68%). Thus, interestingly, we were able to detect both “false” positive ECC and dysplasias with a high chance of having remained undetected if ECC had not been performed.

The discrepancies between the ECC and the histological result after excision may not be true “false” positives, but can also be explained by the fact that curettage can theoretically remove an isolated occult endocervical lesion completely. This may especially be true when there was no visible lesion at the time of colposcopy [18]. Another possible explanation is that the curettage causes an inflammatory reaction leading to increased immune recognition [19]. Alternatively, the lesion may have been missed in the LEEP due to thermal artifact, technical factors, or margin-related issues. We found only a few previous studies evaluating the accuracy of ECC. Müller et al. found a plain agreement rate between ECC and the histological result after excision of 49%. The group of patients who received “*ECC-only*” comprised only 13 patients and the agreement rate was 69.2% in these cases. In our cohort, the “ECC-only” group was larger (*N* = 78), representing 38% of the whole study population and therefore more reliable.

Recently, the endocervical brush and endocervical curettage, as two commonly used methods for obtaining an endocervical or cervical canal sample, were compared in a meta-analysis [20]. The main perspective of the included studies was to compare the two methods, but data on the accuracy of the ECC can be derived from several included studies. Although the number of patients receiving an excisional procedure was lower in all included studies, the reported detection rate of the ECC was in the range of previous reports [21,22,23].

A total of 19 patients in the present study had CIN1 as a result of the ECC. Of these patients, 14 (73.7%) had a CIN2+ as a result of the loop-excisional procedure. This is quite remarkable in our opinion and underlines the fact that CIN1 in the ECC should be taken seriously, as it hints toward an endocervical dysplasia.

We found higher agreement rates in the “ECC + biopsy” group than in the “ECC-only” group. This may be attributed to the effect that the more biopsies are taken, the more dysplasias are detected, which has been shown before [24]. Thus, Pretorius et al. were able to show that the sensitivity of colposcopy is increased by performing more biopsies. In the “ECC-only” group, 35.6% of the patients had a positive ECC and a negative result in the LEEP. This indicates that in this particular group, many patients were diagnosed with CIN2+ via ECC, but it could not be confirmed by LEEP. We hypothesize that the reason is the mechanical alteration of the tissue during the curettage. Often, the tissue is dissolved from the basal membrane, complicating histopathological evaluation. In cases of additionally taken biopsies, only 13.6% had a positive ECC with a negative result of the LEEP.

In the multivariate analysis, the result of colposcopy and the transformation zone, especially combined (kappa 0.259, *p* = 0.0004) had the highest predictive value with regard to the CIN status. So, in case of (partial) visibility of the transformation zone and colposcopic signs for a major change lesion, the best prediction is possible. Unfortunately, with the aging population globally, T3 transformation zones with limited assessability will be a persisting problem in the forthcoming decades, and ECC will not be easily replaced in clinical practice. We recommend keeping the high rate of CIN2+ in mind when counseling patients with CIN1 in the ECC.

Strengths and limitations:

The presented study has strengths and limitations that need to be addressed. First of all, it is the largest available study of ECCs with a high number of available histologies of corresponding excisional procedures using advanced statistical modeling. The subgroup analysis of patients with *ECC-only* is especially relevant to practice regarding the rising number of colposcopies in women with a T3 transformation zone. The calculation of Cohen’s Kappa is also an advantage since it shows the agreement between two raters or methods, while correcting for random agreement. Regarding the limitations, it is important to mention that an analysis of sensitivity and specificity was not possible, as only patients with positive ECC were included in the study. Secondly, we report on a selected cohort of patients seen at a colposcopy unit. Since not all patients routinely underwent ECC, this may introduce a potential bias of the cohort compared to the general population.

## 5. Conclusions

ECC is associated with a low agreement rate in comparison to the final histological result of the LEEP, which should be kept in mind when counseling patients. A finding of CIN1 in an ECC obtained during colposcopy following abnormal HPV-based cervical cancer screening results should be considered an indication of a possible intracervical dysplasia.

## Figures and Tables

**Table 1:** Patients’ characteristics (*N* = 202).

	*N*	%
Age		
Median	41	
Mean ± SD	43.61 ± 11.02	
Min–Max	23–81	
Smoking		
Yes	122	60.4%
No	35	17.3%
Unknown	45	22.3%
Cytology upon referral		
PAP I/II	32	15.8%
PAP IIID1	26	12.9%
PAP IIID2/p/g	61	30.2%
PAP IVa	65	32.2%
PAP IVb/V	14	6.9%
NA	4	2.0%
Control cytology		
PAPI/II	37	18.3%
PAP IIID1	30	14.9%
PAP IIID2/p	67	33.2%
PAP IVap	54	26.7%
PAP IVbp/V	14	6.9%
Colposcopy		
Minor change	55	27.2%
Major change	114	56.4%
Normal	33	16.3%
Localization of transformation zone		
T1	41	20.3%
T2	59	29.2%
T3	102	50.5%
HPV low risk infection		
Yes	59	29.2%
No	139	68.8%
Unknown	4	2.0%
History of HPV		
Category 1	192	95.0%
Category 2B	3	1.5%
No history	4	2.0%
Unknown	3	1.5%
HPV High-risk multiple infection		
Yes	74	36.6%
No	125	61.9%
Missing	3	1.5%
Result of colposcopy-directed ECC		
CINI	19	9.4%
CINII	49	24.3%
CINIII	134	66.3%
Result of colposcopy-directed biopsies		
CINI	16	7.9%
CINII	32	15.8%
CINIII	87	43.1%
Dysplasia free	30	14.9%
Not done	37	18.3%
Result of LEEP		
CINI	24	11.9%
CINII	37	18.3%
CINIII	119	58.9%
Dysplasia free	22	11.0%

**Table 2:** Results of the bivariate statistical analysis using CIN2+ (result of LEEP) as positive endpoint (total *N* = 202).

	Negative		Positive (CIN2+)	
	*N*	%	*N*	%
Total	46	22.8%	156	77.2%
Age				
Mean ± SD	46.0 ± 12		42.8 ± 10	
Colposcopy				
Major change	15	13.2%	99	86.8%
Minor change	18	32.7%	37	67.2%
Normal	13	39.3%	20	60.6%
Smoking				
Yes	30	24.6%	92	75.4%
No	7	20.0%	28	80.0%
Unknown	9	20.0%	36	80.0%
Localization of transformation zone				
T1	7	17.1%	34	82.9%
T2	10	17.0%	49	83.1%
T3	29	28.4%	73	71.6%
Cytology upon referral				
PAP I/II	10	31.3%	22	68.6%
PAP IIID1	8	30.8%	18	69.2%
PAP IIID2/p/g	13	21.3%		
PAP IV-a	12	18.5%	53	81.5%
PAP IV-b/V	2	14.3%	12	85.7%
N missing	1	25%	3	75%
Control cytology				
PAP I/II	8	21.6%	29	78.4%
PAP IIID1	10	33.3%	20	66.7%
PAP IIID2/p	17	25.4%	50	74.6%
PAP IV-a	10	18.52%	44	81.5%
PAP IV-b/V	1	71.4%	13	92.9%
HPV low risk infection				
Yes	14	23.7%	45	76.3%
No	32	23%	107	77%
Unknown	0	0.0%	4	1.0%
HPV high risk infection				
Category 1	41	21.4%	151	78.7%
Category 2B	1	33.3%	2	66.7%
No history	4	1.0%	0	0.0%
Unknown	0	0.0%	3	1.0%
HPV high-risk multiple infection				
Yes	18	24.3%	56	75.7%
No	28	22.4%	97	77.6%
Unknown	0	0.0%	3	1.0%

**Table 3:** Agreement of ECC (endocervical curettage) and result of LEEP (loop-excisional procedure of the cervix) (total *N* = 202).

		**Result of LEEP, *N* (%)**			
		CINI	CINII	CINIII	dysplasia free
Result of ECC, *N* (%)	CINI	3 (15.8%)	5 (26.3%)	9(47.4%)	2 (10.%)
	CINII	9 (18.4%)	17 (34.7%)	17 (34.7%)	6 (12.2%)
	CINIII	12 (8.9%)	15 (11.2%)	93 (69.4%)	14 (10.5%)
		**Result of LEEP, *N* (%)**			
		neg		pos (CIN2+)	
Result of ECC, *N* (%)	CINI	5 (26.3%)		14 (73.7%)	
	CINII	15 (30.6%)		34 (69.4%)	
	CINIII	26 (19.4%)		108 (80.6%)	

**Table 4:** Analysis of the two subgroups regarding the accordance with the histological result of the excisional procedure.

**Subgroup “ECC-Only” Group, *N* = 83**				
		Result of LEEP, *N* (%)		
		Negative (CIN1, Dysplasia Free)	Positive (CIN2+)	Total
Result of ECC, *N* (%)	CIN1	5 (50%)	5 (50%)	10
	CIN2+	26 (35.6%)	47 (65.4%)	73
	Total	31	52	83
**Subgroup “ECC + Biopsy” Group *N* = 119**				
		Result of LEEP, *N* (%)		
		Negative (CIN1, dysplasia free)	Positive (CIN2+)	Total
Result of ECC, *N* (%)	CIN1	0 (0.0%)	9 (100%)	9
	CIN2+	15 (13.6%)	95 (86.4%)	110
	Total	15	104	119

## Data Availability

The data presented in this study are available on request from the corresponding author due to privacy restrictions.

## Abbreviations

The following abbreviations are used in this manuscript:

ECC	Endocervical curettage
CIN	Cervical intraepithelial neoplasia
LEEP	loop-excisional procedure of the cervix
hrHPV	high-risk human papillomavirus
DNA	Deoxyribonucleic Acid
HSIL	high-grade squamous lesions
NILM	Negative for Intraepithelial Lesion or Malignancy
TZ	transformation zone
T3	Transformation zone type 3
LSIL	Low-grade Squamous Intraepithelial Lesion
ASC-H	Atypical Squamous Cells, cannot exclude High-grade squamous intraepithelial lesion
AGC	Atypical Glandular Cells
IACR	International Association of Cancer Registries
IFCPC	International Federation for Cervical Pathology and Colposcopy
AG-CPC	Arbeitsgemeinschaft für Zervixpathologie und Kolposkopie e.V
DKG	Deutsche Krebsgesellschaft
RWTH	Rheinisch-Westfälische Technische Hochschule
SD	standard deviation
Min	minimum
Max	maximum
LOOCV	leave-one-out cross-validation
